# A Sociodemographic Analysis of the Impact of COVID-19-Related Schools’ Closure on the Diet and Physical Activity of Children and Adolescents in Qatar

**DOI:** 10.1007/s44197-023-00101-8

**Published:** 2023-05-04

**Authors:** Muna Abed Alah, Sami Abdeen, Nagah Selim, Elias Tayar, Ayman Al-Dahshan, Vahe Kehyayan, Layla AlDahnaim, Iheb Bougmiza

**Affiliations:** 1 Community Medicine Department, Hamad Medical Corporation (HMC), Doha, Qatar; 2 Community Medicine Department, Primary Health Care Corporation (PHCC), Doha, Qatar; 3 Public Health and Preventive Medicine Department, Cairo University, Giza, Egypt; 4 Healthcare Administration Department, College of Business Management, University of Doha for Science and Technology, Doha, Qatar; 5 School Health Services and Programs, Primary Health Care Corporation (PHCC), Doha, Qatar; 6 Community Medicine Department, College of Medicine, Sousse University, Sousse, Tunisia

**Keywords:** Diet, Physical activity, Children, Adolescents, COVID-19, Schools’ closure

## Abstract

**Objectives:**

To assess the impact of the COVID-19-related closure of government schools in Qatar on children and adolescents' dietary habits and physical activities and associated sociodemographic factors.

**Methods:**

An analytical cross-sectional study was conducted between June and August 2022 utilizing the national electronic health records system in Qatar to extract a sampling frame of students enrolled in governmental schools, specifically targeting students in 3rd to 9th grades, stratified by sex and developmental stage. A stratified sampling technique was employed to randomly select a proportionate number of students from each stratum, and data were collected through telephone interviews with the parents of selected students.

**Results:**

A total of 1546 interviews were completed by the end of the study. Of the included sample, 845 (54.7%) were between 8 and 11 years of age (middle childhood), while the rest were 12–15 years old (young teens and teenagers). Male to female ratio was almost 1:1. We found a significant decrease in the intake of vegetables, increases in the intake of soft drinks, fried food, fast food, and sweets, and a reduction in physical activity during schools’ closure compared to before. Higher parental educational levels, maternal employment, and having a positive family history of obesity and/or overweight in first-degree relatives were significantly associated with adverse lifestyle changes during schools’ closure.

**Conclusion:**

The trends of lifestyle changes reported in this study during the periods of COVID-19-related schools’ closure were found to be going in a health-compromising direction. These results underscore the importance of implementing targeted interventions to promote healthy lifestyles during such disruptions and emphasize the need to address lifestyle changes beyond emergencies and outbreaks to mitigate potential long-term health consequences, including the increased risk of non-communicable diseases.

**Supplementary Information:**

The online version contains supplementary material available at 10.1007/s44197-023-00101-8.

## Introduction

Despite the success of the restrictive precautionary measures imposed by countries worldwide such as the closure of malls, schools, and movement restrictions in containing the spread of COVID-19 infection, the indirect negative repercussions of such measures could not be ignored [1]. The severity and mortality of COVID-19 infection were less among children and adolescents [2, 3]. However, they were not spared from the indirect effects of home confinement measures on their lifestyle [4–7]. A sizable body of evidence has shown a reduction in physical activity and an increase in sedentary behaviors and unhealthy dietary habits among children and adolescents during periods of lockdown and school closures [6–10]. An international study conducted among adolescents aged 10 to 19 years from several regions of Spain, Italy, Brazil, Colombia, and Chile showed a significant increase in the consumption of fried food and an increase in the proportion of adolescents consuming sweet foods every day from 14% to over 20% during lockdown [11]. Regionally, a study in Saudi Arabia reported that about 40% of children and adolescents had difficulty maintaining a healthy, balanced diet during the pandemic, including an increase in the consumption of simple carbohydrates, fried foods, and soft drinks [12]. Regarding the changes in physical activity and sedentary behaviors, a recently published systematic review that included data from 71 studies from 35 countries showed reduced physical activity among children during COVID-19-related lockdown measures [13]. Similar results were also reported in the region in each of Saudi Arabia, Jordan, Palestine, and Tunisia [6, 8, 14, 15]. It is well established in the literature, that unhealthy dietary behaviors such as consuming junk food, sugary drinks, and unhealthy snacks are contributing factors to childhood obesity [16]. Moreover, deleterious metabolic and musculoskeletal implications can be brought about by physical inactivity which can alter glucose and insulin metabolism and impair skeletal muscle protein synthesis [17, 18].

Qatar announced the closure of schools in March 2020 as a step to contain the spread of COVID-19 and to ensure the safety of all students and school staff. Later, some gradual easing of restrictions that involved the partial opening of schools occurred. However, schools never became fully operational with full student capacities until September 2021. To our knowledge, only one study in Qatar assessed the impact of COVID-19-related home confinement measures on the diet and physical activity of children 5–12 years of age and it had limitations because of the small sample size (only 144 participants), the sampling technique used, and the limited definitions of the outcomes measured [19]. This study is part of a larger national research project that assessed the impact of COVID-19-related home confinement and schools’ closure on several lifestyle aspects, vision, and mental health of children and adolescents in Qatar. In this study, we conducted a sociodemographic analysis of the impact of COVID-19-related schools’ closure on the dietary habits and physical activity of children and adolescents in governmental schools in Qatar. Adverse lifestyle changes during school closures, such as unhealthy dietary habits and reduced physical activity, can have long-term health consequences, including the potential for increased risk of non-communicable diseases, such as obesity and related chronic conditions. Understanding the sociodemographic predictors of adverse lifestyle changes can help identify vulnerable populations who may be at a higher risk of experiencing negative health outcomes during school closures. This knowledge can inform the development of targeted interventions that are tailored to the specific needs and challenges of these populations, maximizing their effectiveness in promoting healthy lifestyles.

## Methods

### Study Design and the Target Population

We conducted an analytical cross-sectional study between June and August 2022 targeting students (8–15 years old) registered at governmental schools in Qatar. Students of all nationalities were included.

### Study Procedure and Sampling Technique

Since all students at the governmental schools in Qatar are registered in the national electronic health records system, we used it to extract the sampling frame. First, we asked the Health Information Management section at the Primary Health Care Corporation (PHCC) to extract a list of all students registered at governmental schools between 3rd to 9th grades and stratify them by sex and developmental stage (middle childhood (8–11 years), teens and teenagers (12–15 years)). Second, using a stratified sampling technique, we randomly selected a proportionate number of students from each stratum. The data were collected by conducting telephone interviews with the parents of selected students and verbal consent were obtained and documented.

### Data Collection

#### Data Collection Tool Development and Validation

We developed a questionnaire from multiple tools in English [20, 21]. Then, we translated it into Arabic by an accredited translation body. We piloted both versions to 10 individuals who were selected conveniently and were parents of children of the target age group (8–15 years). We discussed with them the comprehensiveness, language, and grammar used in the questions. We assessed the face, and content validities of the questionnaire by distributing it to six reviewers (three lifestyle medicine board-certified specialists, two experts with experience in running lifestyle medicine clinics, and one nutritionist), and then we asked them to independently rate each item in the questionnaire on a four-point Likert scale as 1(Not relevant), 2 (Somewhat relevant), 3 (Quite relevant), 4 (Highly relevant), and to add their comments on readability, comprehensiveness, clarity, language, and grammar used. Then, the item level content validity index (I-CVI) for each item, and the overall scale content validity index (S-CVI) were calculated. Analysis of the results showed satisfactorily and accepted content validity.

#### Description of the Data Collection Tool

The questionnaire is composed of three sections; the first section was developed through an extensive literature review, and it addresses the sociodemographic characteristics and background information such as age and nationality of the child/adolescent, age of the mother, age of the father, school grade of the child, highest educational level of the mother and father, mother’s employment status, and other background information. The second and third sections address the dietary behaviors and physical activity before and during schools’ closure, respectively. These sections were adapted from existing validated short form survey instruments for children’s diet, physical activity, and sedentary behaviors developed based on the recommendations of the Sax institute [20, 21]. Several dietary behaviors of children and adolescents were assessed by asking parents to indicate the frequency of consumption of different food categories of their children in two periods (before and during COVID-19-related schools’ closure). The definition of a serving and the possible frequency choices for food categories were formulated based on international and local recommendations. To assess physical activity, parents were asked to approximately indicate the number of days in a typical week (from 0 to 7 days) their child did a total of 60 min or more of physical activity, which was enough to raise his/her breathing rate; physical activity may include sport, exercise, brisk walking, cycling for recreation or to get to and from places, or active playing during two periods (before and during schools’ closure). They were also asked to indicate if their child used to practice any type of sports on regular bases before and during the closures. Refer to the supplementary material for the English and Arabic versions of the questionnaires.

### Outcome Measures

We measured the parents' perceived changes in their children's dietary behaviors, and physical activity. The dietary behavioral changes were measured as follows: the changes in the intake of fruits and vegetables were measured as categorical ordinal variables before and during schools’ closure using a three-point measurement scale for fruits: 0 (< 1 serving/day), 1(1–2 servings/day), and 2 (> 2 servings per day), and a four-point measurement scale for vegetables: 0 (< 1 serving/day), 1(1–3 servings/day), 2(4–6 servings/day), and 3(> 6 servings/day). Those who shifted to a lower amount category during schools’ closure were labeled as having “decreased intake”. Those who shifted to a higher amount category were labeled as “increased intake”, and the rest as “stayed the same/no change”. The frequencies of consumption of unhealthy food categories were also measured as categorical ordinal variables before and during schools’ closure on six-point measurement scales: 0 (my child does not drink these drinks), 1 (< 1 cup/week), 2 (1–3 cups/week), 3 (4–6 cups/week), 4 (1–2 cups/days), 5 (3 or more cups/day), for soft drinks/sweetened beverages/energy drinks and on a seven, point measurement scale: 0 (never or rarely), 1 (< 1 time/week), 2 (1–2 times/week), 3 (3–4 times/week), 4 (5–6 times/week), 5 (about 1 time /day), 6 (2 or more times/day) for fried food prepared at home, junk/fast food prepared at fast food restaurants, and sugar-based sweets such as candies, chocolate, and jam. Physical activity was measured as a quantitative discrete variable (from 0 to 7 days) in a typical week before and during schools’ closure. The change in physical activity was calculated based on the difference in the days of physical activity during closure compared to before closure (number of days during-number of days before). A positive value was labeled as “increased physical activity”, while a negative value as “decreased”, and zero as “no change”. We also reported the percentage of participants meeting the World Health Organization’s (WHO) physical activity recommendations of practicing at least 60 min per day of moderate to vigorous intensity physical activity across the week before and during schools’ closure [22].

### Statistical Analysis

The data were analyzed using IBM SPSS Statistics for Windows, Version 26.0. Armonk, NY: IBM Corp. For descriptive statistics, we used percentages for categorical data while mean and standard deviation for numerical data. For univariate analysis, we used the Chi-square or Fisher Exact tests as appropriate for categorical variables and the independent Student’s *t* test or Mann–Whitney *U* test as appropriate for continuous and ordinal variables. In addition, the Wilcoxon Signed Rank test was used to compare the frequency of intake of each of the food categories and the frequency of physical activity before and during school closures in the total sample and both sexes and age groups. McNemar's test was used to compare the percentage of children and adolescents meeting the WHO’s physical activity recommendations during closure compared to before closure. To determine the potential predictors for lifestyle changes, logistic regression analyses were performed. The associations between risk factors and outcomes were presented as adjusted odds ratios (AORs) and 95% confidence intervals (95%CIs). The goodness of Fit was assessed using the Hosmer–Lemeshow test. *P* values less than 0.05 were considered significant.

## Results

### Sociodemographic Characteristics and Background Information of the Included Children and Adolescents

A total of 1546 completed questionnaires out of 3327 approaches were collected during the telephone interviews with parents of selected children and adolescents between 8 and 15 years of age giving a response rate of about 46%. According to the Centers for Disease Control and Prevention (CDC), those between 8 and 11 were considered in the Middle childhood stage, those between 12 and 14 were considered young teens, and those who were 15 years old were considered teenagers [23]. As shown in Table 1, 845 (54.7%) were between 8 and 11 years of age (middle childhood) while the rest were 12–15 years old (young teens and teenagers). The proportion of females and males was almost the same (females: 777, 50.3%), and (males: 769, 49.7%). The majority (1377, 89.1%) were Arabs, with Qatari nationality being the top reported (572, 37%). Of the participants, 588 (38%) had a positive family history of obesity or overweight in one or more of first-degree relatives. Only 8% of the interviewed parents disclosed their child’s diagnosis with a chronic disease with asthma being the commonest reported diagnosis (81 out of 1546, 5.2%).

**Table 1:** Sociodemographic characteristics and related background information

Characteristic	Sex		*p* value^c^	Developmental stage		*p* value^c^	Total *N = *1546 M ± SD no (%)
	Female *n = *777 M ± SD no (%)	Male *n = *769 M ± SD no (%)		Middle childhood *n = *845 M ± SD no (%)	Teens and teenagers *n = *701 M ± SD No (%)		
Student age	11 ± 2	11 ± 2	**0.036**	10 ± 1	13 ± 1	** <0.001**	11 ± 2
Developmental stage							
Middle childhood (8–11 years)	415 (35.4)	430 (55.9)	0.322	–	–	–	845 (54.7)
Teens and teenagers (12–15 years)	362 (46.6)	339 (44.1)		–	–		701 (45.3)
Sex							
Female	–	–	–	415 (9.1)	362 (51.6)	0.322	777 (50.3)
Male	–	–		430 (50.9)	339 (48.4)		769 (49.7)
Nationality^a^ (Qatari, non-Qatari)							
Non-Qatari	507 (65.3)	467 (60.7)		537 (63.6)	437 (62.3)		974 (63.0)
Qatari	270 (34.7)	302 (39.3)		308 (36.4)	264 (37.7)		572 (37.0)
Number of siblings							
3 or less	354 (45.6)	386 (50.2)	**0.046**	431 (51.0)	309 (44.1)	**0.001**	740 (47.9)
4–6	339 (43.6)	316 (41.1)		348 (41.2)	307 (43.8)		655 (42.4)
> 6	84 (10.8)	67 (8.7)		66 (7.8)	85 (12.1)		151 (9.8)
Family history of obesity							
No	509 (65.5)	449 (58.4)	**0.004**	550 (65.1)	408 (58.2)	**0.005**	958 (62.0)
Yes	268 (34.5)	320 (41.6)		295 (34.9)	293 (41.8)		588 (38.0)
Chronic disease^b^							
No	717 (92.3)	706 (91.8)	0.733	786 (93.0)	637 (90.9)	0.120	1423 (92.0)
Yes	60 (7.7)	63 (8.2)		59 (7.0)	64 (9.1)		123 (8.0)
Family income							
Less than 10,000 QR	125 (16.1)	64 (8.3)	0.094	107 (12.7)	82 (11.7)	0.189	189 (12.2)
10,000–30,000 QR	184 (23.7)	94 (12.2)		133 (15.7)	145 (20.7)		278 (18.0)
30,000–50,000 QR	30 (3.9)	18 (2.3)		25 (3.0)	23 (3.3)		48 (3.1)
More than 50,000 QR	14 (1.8)	21 (2.7)		18 (2.1)	17 (2.4)		35 (2.3)
Don't want to answer	424 (54.6)	572 (74.4)		562 (66.5)	434 (61.9)		996 (64.4)
Sociodemographic characteristics of parents							
Mother age	40 ± 6	39 ± 6	0.313	38 ± 6	41 ± 6	** < 0.001**	40 ± 6
Mother age categories							
< 35	144 (18.8)	153 (20.4)	0.244	216 (26.2)	81 (11.8)	** < 0.001**	297 (19.6)
35–44	454 (89.3)	448 (59.8)		489 (59.2)	413 (59.9)		902 (59.5)
45–54	161 (21.0)	142 (19.0)		119 (14.4)	184 (26.7)		303 (20.0)
55 or more	7 (0.6)	6 (0.8)		2 (0.2)	11 (1.6)		13 (0.9)
Father’s age	45 ± 7	45 ± 8	0.766	44 ± 7	47 ± 8	** < 0.001**	45 ± 8
Father’s age categories							
< 35	27 (3.5)	41 (5.5)	0.599	56 (6.8)	12 (1.7)	** < 0.001**	68 (4.5)
35–44	349 (45.7)	339 (45.7)		415 (50.7)	273 (39.8)		688 (45.7)
45–54	305 (40.0)	263 (35.5)		279 (34.1)	289 (42.1)		568 (37.8)
55 or more	82 (10.7)	98 (13.2)		68 (8.3)	112 (16.3)		180 (12.0)
Mother education							
No formal education	43 (5.5)	33 (4.3)	0.536	35 (4.1)	41 (5.8)	0.152	76 (4.9)
Primary school level	56 (7.2)	45 (5.9)		52 (6.2)	49 (7.0)		101 (6.5)
Preparatory school level	65 (8.4)	61 (7.9)		75 (8.9)	51 (7.3)		126 (8.2)
Secondary/ high school level	222 (28.6)	244 (31.7)		244 (28.9)	222 (31.7)		466 (30.1)
College or higher	391 (50.3)	386 (50.2)		439 (52.0)	338 (48.2)		777 (50.3)
Father education							
No formal education	20 (2.6)	20 (2.6)	0.941	21 (2.5)	19 (2.7)	0.180	40 (2.6)
Primary school level	43 (5.5)	34 (4.4)		43 (5.1)	34 (4.9)		77 (5.0)
Preparatory school level	76 (9.8)	61 (7.9)		69 (8.2)	68 (9.7)		137 (8.9)
Secondary/ high school level	200 (25.7)	214 (27.8)		218 (25.8)	196 (28.0)		414 (26.8)
College or higher	438 (56.4)	440 (57.2)		494 (58.5)	384 (54.8)		878 (56.8)
Mother employment							
Employed	338 (43.5)	373 (48.5)	**0.048**	389 (46.0)	322 (45.9)	0.968	711 (46.0)
Not employed	439 (56.5)	396 (51.5)		456 (54.0)	379 (54.1)		835 (54.0)

*M* mean; *SD* standard deviation

^a^28 different nationalities were reported

^b^Most commonly reported chronic diseases were Asthma and Diabetes

^c^Using Chi square or Fisher Exact test as appropriate for categorical variables and independent *t*-test or *t*-test and Mann–Whitney test as appropriate for continuous and ordinal variables. *P* values <0.05 were considered significant and bolded

### Sociodemographic Characteristics of the Parents of Selected Children and Adolescents

By analyzing the data of the parents of included participants, most were between 35 and 44 years of age. The sample appeared highly educated with about half of the parents having a college degree or higher while less than 5% had no formal education. Of the mothers, 711 (46%) were employed at the time of data collection (Table 1).

### The Impact of COVID-19-Related School Closures on the Dietary Habits of Children and Adolescents

#### The Change in the Intake of Fruit and Vegetables

The intake of fruit before and during school closures among most students was reported to be an average of 1–2 servings/day (72.6% before, and 64.2% during) as shown in Table 2. Analyzing the change in fruit intake during the closure showed a statistically non-significant increase in the intake of fruits during school closures compared to before closure (*p* = 0.459) in the total sample.

**Table 2:** Dietary behaviors and physical activity of children and adolescents by sex and developmental stages at two time periods (before and during schools’ closure)

Variable	Sex			Developmental stage		
	Female	Male	*p* value^a^	Middle childhood	Teens and teenagers	*p* value^a^
	Mean rank	Mean rank		Mean rank	Mean rank	
Diet						
Fruit intake						
Before closure	792	755	**0.033**	748	804	**0.001**
During closure	804	742	**0.001**	732	824	** < 0.001**
Vegetable intake						
Before closure	784	763	0.211	770	777	0.686
During closure	781	766	0.405	765	784	0.292
Soft drinks intake						
Before closure	847	700	** < 0.001**	715	844	** < 0.001**
During closure	782	764	0.408	714	844	** < 0.001**
Home-fried food intake						
Before closure	769	778	0.633	791	753	0.059
During closure	809	737	**0.001**	771	777	0.789
Junk food intake						
Before closure	761	786	0.234	797	745	**0.010**
During closure	794	753	0.064	761	789	0.195
Sweets intake						
Before closure	813	733	** < 0.001**	769	778	0.678
During closure	793	753	0.070	773	774	0.952
Physical activity						
Physical activity^b^						
Before closure	677	871	** < 0.001**	790	753	0.094
During closure	684	864	** < 0.001**	819	718	** < 0.001**

*P* values <0.05 were considered significant and bolded

^a^Using Mann–Whitney test

^b^No of days a in a regular week with 60 + minutes of physical activity

Upon assessing the intake of vegetables before and during school closures, we found it among most students to be on an average of 1–3 servings/day (76.3% before, and 72.3% during). No significant differences were found in the intake between the sexes or between different developmental stages (Table 2). There was a significant decrease in the intake of vegetables during school closures compared to before in the total sample (*P* < 0.001) (Table 3).

**Table 3:** The changes in dietary behaviors and physical activity during COVID-19-related schools’ closure in the total sample, both sexes, and developmental stages

Variable	Sex										Developmental stage										Total				
	Female					Male					Middle Childhood					Teens and teenagers									
	N	Mean rank	Sum of ranks	*p* value^a^	*r*	*N*	Mean rank	Sum of ranks	*p* value^a^	*r*	*N*	Mean rank	Sum of ranks	*p* value^a^	*r*	*N*	Mean rank	Sum of ranks	*p* value^a^	*r*	*N*	Mean rank	Sum of ranks	*p* value^a^	*r*
Diet																									
Fruit intake																									
Negative ranks	54	64	3429	0.079	–	50	46	2300	0.345	–	53	56	2968	0.635	–	51	54	2728	0.567	–	104	109	11,336	0.459	–
Positive ranks	73	64	4699			41	46	1886			58	56	3248			56	54	3049			114	110	12,535		
Vegetable intake																									
Negative ranks	42	32	1323	**0.005**	-0.4	42	32	1344	**0.008**	-0.3	44	3	1628	0.079	–	40	27	1060	** < 0.001**	-0.5	84	63	5292	** < 0.001**	-0.3
Positive ranks	20	32	630			21	32	672			29	37	1073			12	27	318			41	63	2583		
Soft drinks intake																									
Negative ranks	106	109	11,601	0.374	–	40	127	5119	** < 0.001**	0.4	89	129	11,566	0.767	–	57	93	5305	**0.001**	0.3	146	224	32,711	**0.021**	0.1
Positive ranks	102	99	10,135			139	79	10,991			128	94	12,086			113	82	9229			241	176	42,366		
Home-fried food intak																									
Negative ranks	20	101	2011	** < 0.001**	0.8	18	138	2482	** < 0.001**	0.8	20	151	3028	** < 0.001**	0.8	18	87	1572	** < 0.001**	0.8	38	236	8974.5	** < 0.001**	0.8
Positive ranks	186	103	19,310			190	101	19,253			229	122	28,097			147	82	12,123			376	205	76,930.5		
Junk food intake																									
Negative ranks	126	159	20,108.5	0.090	–	83	183	15,146	** < 0.001**	3.5	117	180	21,169	0.327	–	92	165	15,215	**0.045**	0.1	209	345	72,120.5	**0.035**	0.1
Positive ranks	142	112	15,937.5			214	136	29,107			183	131	23,980			173	116	20,029			356	247	87,774.5		
Sweets intake																									
Negative ranks	25	114	2860	** < 0.001**	0.8	15	194	2910	** < 0.001**	0.9	24	171	4109	** < 0.001**	0.9	16	126	2014	** < 0.001**	0.8	40	299	11,969.5	** < 0.001**	0.8
Positive ranks	216	121	26,301			308	160	49,415			330	177	58,726			194	104	20,141			524	281	147,360.5		
Physical activity																									
Physical activity																									
Negative ranks	419	225	94,126	** < 0.001**	− 0.7	317	180	56,917	** < 0.001**	− 0.7	368	205	75,446	** < 0.001**	− 0.7	368	198	72,845	** < 0.001**	0.8	736	402	296,133	** < 0.001**	− 0.7
Positive ranks	48	316	15,152			48	206	9877			59	270	15,932			37	253	9370			96	525	50,395		

*P *values <0.05 were considered significant and bolded

*r* rank-biserial correlation coefficient

^a^Using Wilcoxon signed rank test. Negative ranks indicate decreased intake during closure and positive ranks indicate increased intake during closure

#### The Change in the Frequency of Intake of Soft Drinks/Sweetened Beverages

The frequency of intake of soft drinks/sweetened beverages before and during schools’ closure among most students was on average 1–3 cups/week (36.4% before, and 41% during). As shown in Table 3, we found a significant increase in the intake of these beverages during schools’ closure compared to before in the total sample (*P* < 0.021).

#### The Change in the Frequency of Intake of Fried Food (Prepared at Home), and Junk Food (Prepared at Fast Food Restaurants)

The frequency of the intake of fried food that is prepared at home before and during schools’ closure among most students was on average 1–2 times/week (60.3% before and 51.6% during) (Table 2). We found a significant increase in the frequency of intake of fried food that was prepared at home during schools’ closure compared to before closure in the total sample (*P* < 0.001).

Regarding the frequency of intake of Junk/fast food from fast food restaurants before and during schools’ closure, it was mostly reported to be on an average of 1–2 times/week (54.7% before, and 40.3% during), and there was a significant increase in the frequency of intake during closure compared to before in the total sample (*P* = 0.035) as shown in Table 3.

#### The Change in the Intake of Sugar-Based Sweets

The frequency of the intake of sugar-based sweets was mostly reported to be on an average of 1–2 times/week before closure (47.5%) and 3–4 times/week during closure (30.9%). There was a significant increase in the frequency of sugar-based sweets intake during schools’ closure in the total sample which was also significant among different sexes and developmental stages (*P* < 0.001) (Table 3).

### The Impact of COVID-19-Related School Closures on the Physical Activity of Children and Adolescents

Most children and adolescents (88.7%) were not meeting the WHO’s physical activity recommendations of practicing moderate to vigorous physical activity for 60 min/per day across the week before schools’ closure. This percentage further increased during closure to reach 90.4%. Most of the included participants (66.8%) used to practice physical activity on an average of 2–4 days/week before closure, and this percentage was halved during closure down to 32%. Significantly higher proportions of males and younger children were meeting WHO’s recommendations before and during closure than females and older children as shown in Table 2. About a quarter of participants used to practice sports regularly outside schools before closure and this percentage dropped to less than 5% during closure.

Analyzing the change in physical activity, we found a significant reduction in the frequency of practicing physical activity during schools’ closure in the total sample, in both sexes, and in all developmental stages with *P* values of < 0.001 (Table 3). A subgroup analysis using McNemar’s test showed a significant drop in the proportion of those meeting the WHO’s recommendations among males (from 16.3 to 13.9%, *P* = 0.041), and among teens and teenagers of 12–15 years of age (9–5.8%, *P* < 0.001). We also found a significant drop in the proportion of children and adolescents practicing sports regularly by 20% (from 23.1% before to 2.8% during, *P* < 0.001).

### Sociodemographic Determinants and Predictors of Adverse Dietary Changes During COVID-19-Related School Closures

#### Determinants and Predictors of the Reduction in the Intake of Healthy Food

Table 4 shows the results of the univariable analysis of comparing the change in the intake of healthy food (fruit and vegetables) among different sociodemographic groups.

**Table 4:** The determinants of adverse dietary changes in healthy food, and physical activity during COVID-19-related schools’ closure using univariate analysis

Participants’ characteristic	Changes in Diet				Change in PA	
	Decreased fruit intake no (%)	*p* value^a^	Decreased Vegetable intake no (%)	*p* value^a^	Decreased PA time no (%)	*p* value^a^
Developmental stage						
Middle childhood (8–11 years)	53 (6.3)	0.433	44 (5.2)	0.667	360 (42.6)	** < 0.001**
Teens and teenagers (12–15 years)	51 (7.3)		40 (5.7)		367 (52.4)	
Sex						
Female	54 (6.9)	0.725	42 (5.4)	0.961	407 (52.4)	** < 0.001**
Male	50 (6.5)		42 (5.5)		320 (41.6)	
Nationality (Qatari, non-Qatari)						
Non-Qatari	61 (6.3)	0.342	49 (5.0)	0.362	501 (51.4)	** < 0.001**
Qatari	43 (7.5)		35 (6.1)		226 (39.5)	
Number of siblings						
3 or less	48 (6.5)	0.811	44 (5.9)	0.264	390 (52.7)	** < 0.001**
4–6	47 (7.2)		36 (5.5)		286 (43.7)	
> 6	9 (6.0)		4 (2.6)		51 (33.8)	
Family history of obesity						
No	54 (5.6)	**0.029**	47 (4.9)	0.243	445 (46.5)	** < 0.001**
Yes	50 (8.5)		37 (6.3)		282 (48.0)	
Chronic disease						
No	95 (6.7)	0.785	75 (5.3)	0.337	661 (46.5)	0.124
Yes	9 (7.3)		9 (7.3)		66 (53.7)	
Mother age categories						
< 35	20 (6.7)	0.906	16 (5.4)	0.685	154 (51.9)	0.244
35–44	57 (6.3)		44 (4.9)		421 (46.7)	
45–54	23 (7.6)		20 (6.6)		137 (45.2)	
55 or more	1 (7.7)		1 (7.7)		8 (61.5)	
Father age categories						
< 35	6 (8.8)	0.269	5 (7.4)	0.120	32 (47.1)	0.877
35–44	43 (6.3)		32 (4.7)		332 (48.3)	
45–54	32 (5.6)		24 (4.2)		266 (46.8)	
55 or more	17 (9.4)		15 (8.3)		81 (45.0)	
Mother education						
No formal education	3 (3.9)	0.355	2 (2.6)	**0.013**	24 (31.6)	** < 0.001**
Primary school level	6 (5.9)		3 (3.0)		43 (42.6)	
Preparatory school level	6 (4.8)		2 (1.6)		47 (37.3)	
Secondary/ high school level	27 (5.8)		20 (4.3)		202 (43.3)	
College or higher	62 (8.0)		57 (7.3)		411 (52.9)	
Father education						
No formal education	3 (7.5)	0.114	2 (5.0)	**0.017**	13 (32.5)	** < 0.001**
Primary school level	4 (5.2)		3 (3.9)		30 (39.0)	
Preparatory school level	9 (6.6)		4 (2.9)		50 (46.5)	
Secondary/ high school level	17 (4.1)		12 (2.9)		181 (34.7)	
College or higher	71 (8.1)		63 (7.2)		453 (51.6)	
Mother employment						
Employed	45 (6.3)	0.564	37 (5.2)	0.713	335 (47.1)	0.947
Not employed	59 (7.1)		47 (5.6)		392 (46.9)	

*PA* physical activity

* P *values <0.05 were considered significant and bolded

^a^Using Chi square or Fisher Exact test as appropriate

Using logistic regression, we found that participants with a family history of obesity and/or overweight were about 1.5 times more likely to decrease their intake of fruits during closure compared to those without (AOR 1.56, 95%CI 1:01, 2.26, *P* = 0.048). However, the regression did not yield any significant independent sociodemographic predictors of the change in vegetable intake.

#### Determinants and Predictors of the Increase in the Frequency of Intake of Unhealthy Food Groups

In Table 5, we summarized the results of univariable analysis by comparing the change (increased vs decreased or no change) in the frequency of intake of unhealthy food (soft drinks/sweetened beverages, fried food, fast/junk food, and sugar-based sweets) between different sociodemographic subgroups.

**Table 5:** The determinants of adverse dietary changes in unhealthy food during COVID-19-related schools’ closure using univariate analysis

Participants’ characteristics	Increased soft drinks intake no (%)	*p* value^a^	Increased home fried food intake No (%)	*p* value^a^	Increased junk food intake no (%)	*p* value^a^	Increased sweet intake no (%)	*p* value*
Developmental stage								
Middle childhood (8–11 years)	128 (15.1)	0.600	229 (27.1)	**0.005**	183 (21.7)	0.160	330 (39.1)	** < 0.001**
Teens and teenagers (12–15 years)	113 (16.1)		147 (21.0)		173 (24.7)		194 (27.7)	
Sex								
Female	102 (13.1)	**0.007**	186 (23.9)	0.724	142 (18.3)	** < 0.001**	216 (27.8)	** < 0.001**
Male	139 (18.1)		190 (24.7)		214 (27.8)		308 (40.1)	
Nationality (Qatari, non-Qatari)								
Non-Qatari	153 (15.7)	0.865	255 (26.2)	**0.026**	195 (20.0)	** < 0.001**	320 (32.9)	0.260
Qatari	88 (15.4)		121 (21.2)		161 (28.1)		204 (35.7)	
Number of siblings								
3 or less	140 (18.9)	**0.002**	212 (28.6)	** < 0.001**	202 (27.3)	**0.001**	303 (40.9)	** < 0.001**
4–6	80 (12.2)		141 (21.5)		126 (19.2)		186 (28.4)	
> 6	21 (13.9)		23 (15.2)		28 (18.5)		35 (23.2)	
Family history of obesity								
No	136 (14.2)	**0.012**	224 (23.4)	0.759	185 (19.3)	** < 0.001**	298 (31.1)	** < 0.001**
Yes	105 (17.9)		152 (25.9)		171 (29.1)		226 (38.4)	
Chronic disease								
No	226 (15.9)	0.280	350 (24.6)	0.391	327 (23.0)	0.880	492 (34.6)	0.054
Yes	15 (12.2)		26 (21.1)		29 (23.6)		32 (26.0)	
Mother age categories								
< 35	37 (12.5)	0.363	66 (22.2)	0.799	56 (18.9)	0.051	87 (29.3)	0.145
35–44	147 (16.3)		220 (24.4)		200 (22.2)		317 (35.1)	
45–54	45 (14.9)		70 (23.1)		82 (27.1)		90 (29.7)	
55 or more	3 (23.1)		4 (30.8)		5 (38.2)		5 (38.5)	
Father age categories								
< 35	5 (7.4)	**0.019**	14 (20.6)	0.068	9 (13.2)	**0.006**	18 (26.5)	0.672
35–44	95 (13.8)		146 (21.2)		148 (21.5)		229 (33.3)	
45–54	95 (16.7)		156 (27.5)		124 (21.8)		193 (34.0)	
55 or more	38 (21.1)		44 (24.4)		57 (31.7)		60 (33.3)	
Mother education								
No formal education	3 (3.9)	** < 0.001**	7 (9.2)	** < 0.001**	5 (6.6)	** < 0.001**	6 (7.9)	** < 0.001**
Primary school level	7 (6.9)		12 (11.9)		7 (6.9)		19 (18.8)	
preparatory school level	13 (10.3)		21 (16.7)		16 (12.7)		28 (22.2)	
Secondary/ high school level	69 (14.8)		100 (21.5)		108 (23.2)		140 (30.0)	
College or higher	149 (19.2)		236 (30.4)		220 (28.3)		331 (42.6)	
Father education								
No formal education	2 (5.0)	** < 0.001**	4 (10.0)	** < 0.001**	5 (12.5)	** < 0.001**	5 (12.5)	** < 0.001**
Primary school level	12 (15.6)		12 (15.6)		16 (20.8)		12 (15.6)	
Preparatory school level	10 (7.3)		15 (10.9)		15 (10.9)		18 (13.1)	
Secondary/ high school level	51 (12.3)		75 (18.1)		76 (18.4)		101 (24.4)	
College or higher	166 (18.9)		270 (30.8)		244 (27.8)		388 (44.2)	
Mother employment								
Employed	122 (17.2)	0.116	196 (27.6)	**0.006**	217 (30.5)	** < 0.001**	306 (43.0)	** < 0.001**
Not employed	119 (14.3)		180 (21.6)		139 (16.6)		218 (26.1)	

*PA* physical activity

*P* values <0.05 were considered significant and bolded

^a^Using Chi square or Fisher Exact test as appropriate

By carrying out four logistic regression models, we found that the sex of the participant, the mother’s educational level, the father’s age group, and the number of siblings were significant predictors of the increase in soft drinks/sweetened beverages intake. Participants whose fathers were 55 years or more, were over three times more likely to increase their intake during closure compared to those whose fathers were 35 years or less (AOR 3.42, 95%CI 1.24, 9.47, *P* = 0.018). On the other hand, females (AOR 0.67, 95%CI 0.50, 0.90, *P* = 0.007), children whose mothers had no formal education (AOR 0.26, 95%CI 0.07, 0.93, *P* = 0.038) were less likely to increase their soft drinks intake during schools’ closure compared to males, and those whose mothers had a college degree or higher, respectively (Table 6). The participant’s developmental stage and father’s educational level were independent predictors of the increase in the frequency of the intake of fried food prepared at home (Table 6).

**Table 6:** Predictors of adverse lifestyle changes using multivariable logistic regression analyses

Characteristics^a^	Increased soft drinks intake		Increased home fried food intake		Increased junk food intake		Increased sweets intake		Decreased physical activity	
	AOR (95% CI)	*P* value^b^	AOR (95% CI)	*P* value^b^	AOR (95% CI)	*P* value^b^	AOR (95% CI)	*P* value^b^	AOR (95% CI)	*P* value^b^
Developmental stage										
Middle childhood (8–11 years)	0.96 (0.72–1.29)	0.790	1.44 (1.12–1.86)	**0.005**	0.84 (0.65–1.10)	0.209	1.74 (1.37–2.21)	** < 0.001**	0.62 (0.50–0.77)	** < 0.001**
Teens and teenagers (12–15 years)	1 [Reference]		1 [Reference]		1 [Reference]		1 [Reference]		1 [Reference]	
Sex										
Female	0.67 (0.50–0.90)	**0.007**	0.98 (0.77–1.26)	0.883	0.62 (0.47–0.80)	** < 0.001**	0.63 (0.50–0.79)	** < 0.001**	1.61 (1.30–1.99)	** < 0.001**
Male	1 [Reference]		1 [Reference]		1 [Reference]		1 [Reference]		1 [Reference]	
Nationality (Qatari, non-Qatari)										
Non-Qatari	0.95 (0.68–1.31)	0.745	1.15 (0.87–1.52)	0.332	0.67 (0.50–0.89)	**0.006**	0.77 (0.59–1.00)	0.051	1.40 (1.11–1.78)	**0.005**
Qatari	1 [Reference]		1 [Reference]		1 [Reference]		1 [Reference]		1 [Reference]	
Number of siblings										
3 or less	1 [Reference]		1 [Reference]		1 [Reference]		1 [Reference]		1 [Reference]	
4–6	0.60 (0.43–0.83)	**0.002**	0.78 (0.59–1.02)	0.064	0.63 (0.47–0.85)	**0.002**	0.67 (0.51–0.86)	**0.002**	1.70 (1.10–2.61)	**0.016**
> 6	0.96 (0.53–1.71)	0.877	0.74 (0.43–1.27)	0.275	0.77 (0.45–1.31)	0.336	0.86 (0.53–1.40)	0.553	1.23 (0.82–1.85)	0.328
Family history of obesity										
No	1 [Reference]		–	–	1 [Reference]		1 [Reference]		1 [Reference]	
Yes	1.21 (0.90–1.64)	0.206	–	–	1.58 (1.21–2.07)	**0.001**	1.50 (1.18–1.91)	**0.001**	1.18 (0.95–1.48)	0.132
Chronic disease										
No	–	–	–	–	1 [Reference]		1 [Reference]		1 [Reference]	
Yes	–	–	–	–	–	–	0.91 (0.58–1.43)	0.691	1.55 (1.04–2.29)	**0.030**
Mother age categories										
< 35	–	–	–	–	–	–	1 [Reference]		1 [Reference]	
35–44	–	–	–	–	1.05 (0.70–1.57)	0.819	1.31 (0.96–1.79)	0.093	0.72 (0.54–0.95)	**0.020**
45–54	–	–	–	–	1.18 (0.69–2.03)	0.544	1.16 (0.78–1.72)	0.469	0.70 (0.49–0.99)	**0.044**
55 or more	–	–	–	–	2.1 (0.55–8.04)	0.273	2.78 (0.78–9.91)	0.115	1.50 (0.46–4.90)	0.506
Father age categories										
< 35	1 [Reference]		1 [Reference]		1 [Reference]		–	–	–	–
35–44	1.91 (0.73–5.00)	0.184	0.93 (0.49–1.76)	0.820	1.77 (0.76–4.09)	0.184	–	–	–	–
45–54	2.36 (0.90–6.22)	0.082	1.39 (0.73–2.66)	0.322	1.71 (0.70–4.14)	0.239	–	–	–	–
55 or more	3.42 (1.24–9.47)	**0.018**	1.49 (0.73–3.04)	0.280	2.67 (1.02–6.96)	0.045	–	–	–	–
Mother education										
No formal education	0.26 (0.07–0.93)	**0.038**	0.52 (0.21–1.30)	0.162	0.42 (0.15–1.20)	0.106	0.41 (0.16–1.05)	0.062	0.47 (0.25–0.89)	**0.020**
Primary school level	0.42 (0.17–1.01)	0.052	0.58 (0.29–1.16)	0.121	0.36 (0.15–0.86)	**0.021**	0.97 (0.53–1.77)	0.923	0.77 (0.47–1.26)	0.302
Preparatory school level	0.65 (0.33–1.29)	0.219	0.81 (0.46–1.41)	0.449	0.74 (0.40–1.39)	0.348	0.98 (0.58–1.66)	0.940	0.66 (0.42–1.04)	0.074
Secondary/ high school level	0.83 (0.57–1.21)	0.329	0.89 (0.65–1.23)	0.480	0.99 (0.71–1.38)	0.944	0.93 (0.69–1.26)	0.641	0.75 (0.57–0.99)	**0.040**
College or higher	1 [Reference]		1 [Reference]		1 [Reference]		1 [Reference]		1 [Reference]	
Father education										
No formal education	0.40 (0.09–1.87)	0.245	0.46 (0.15–1.43)	0.177	0.63 (0.21–1.92)	0.415	0.27 (0.09–0.79)	**0.017**	0.71 (0.31–1.58)	0.398
Primary school level	1.20 (0.58–2.50)	0.620	0.62 (0.31–1.24)	0.176	0.98 (0.49–1.94)	0.949	0.25 (0.12–0.51)	** < 0.001**	0.84 (0.49–1.44)	0.518
Preparatory school level	0.49 (0.24–1.01)	0.052	0.38 (0.21–0.70)	**0.002**	0.45 (0.24–0.83)	**0.010**	0.24 (0.14–0.42)	** < 0.001**	0.65 (0.42–0.99)	**0.046**
Secondary/ high school level	0.72 (0.48–1.07)	0.106	0.62 (0.44–0.87)	**0.005**	0.70 (0.49–0.99)	**0.047**	0.47 (0.34–0.64)	** < 0.001**	0.91 (0.69–1.21)	0.515
College or higher	1 [Reference]		1 [Reference]		1 [Reference]		1 [Reference]		1 [Reference]	
Mother employment										
Employed	0.91 (0.67–1.24)	0.551	1.13 (0.87–1.48)	0.362	1.70 (1.28–2.26)	** < 0.001**	1.55 (1.21–2.00)	**0.001**	0.89 (0.70–1.12)	0.313
Not employed	1 [Reference]		1 [Reference]		1 [Reference]		1 [Reference]		1 [Reference]	

*AOR* adjusted odds ratio; *CI* confidence interval

^a^Variables included in each regression model are those who have a *P*-value < 0.250 in the univariable analysis (from Tables 4 and 5). ^b^*P* values <0.05 were considered signficiant and bolded

The sex, nationality, both parents’ educational levels, father’s age group, mother’s employment status, number of siblings, and family history of obesity and or overweight were significant independent predictors of the increase in junk/fast food intake. Students whose fathers were 45-54 years old (AOR 2.67, 95%CI 1.02, 6.96, *P* = 0.045), had employed mothers (AOR 1.7, 95%CI 1.28, 2.26, *P* < 0.001), and had a positive family history of obesity and/or overweight (AOR: 1.58, 95%CI 1.21, 2.07, *P* = 0.001) were more likely to increase their intake (Table 6).

The study showed that the child’s developmental stage, sex, father’s educational level, mother’s employment status, number of siblings, and family history of obesity and/or overweight were significantly and independently associated with the increase in sweets intake. Younger students were about 1.7 times more likely to report increased intake compared to older students (AOR 1.74, 95%CI 1.37, 2.21, *P* < 0.001), and students whose mothers were employed were about 1.5 times more likely to increase their intake (AOR: 1.55, 95%CI 1.21, 2.00, *P* = 0.001) (Table 6).

### Sociodemographic Determinants and Predictors of Reduced Physical Activity during COVID-19-Related Schools’ Closure

Using the Chi-square test to compare the change in physical activity (decreased Vs increased or stayed the same) among different sociodemographic groups, we found that the student’s developmental stage, sex, nationality, parents’ educational levels, and the number of siblings were significantly associated with the reduction in physical activity as shown in Table 4.

Logistic regression showed that all the above-mentioned variables along with the mother’s age and having a history of chronic diseases like asthma were significant predictors of the reduction in physical activity. Females were about 1.6 times more likely to reduce their physical activities compared to males (AOR 1.61, 95%CI 1.30, 1.99, *P* < 0.001). Non-Qatari students were about 1.4 times more likely to report a reduction in physical activity compared to Qatari students (AOR 1.40, 95%CI 1.11, 1.78, *P* = 0.005). Similarly, students with a history of chronic diseases were about 1.5 times more likely to reduce their physical activity compared to students without (AOR 1.55, 95%CI 1.04, 2.29, *P* = 0.030) as shown in Table 6.

## Discussion

The COVID-19 pandemic has resulted in numerous deleterious implications for the health of people worldwide. The indirect consequences of the pandemic and related containment measures on the lifestyle of people started to come to light and many others are yet to be elucidated. In this study, we assessed the impact of COVID-19-related school closures on the diet and physical activity of children and adolescents in Qatar and their associated sociodemographic determinants and predictors. Looking into the literature and available studies, it was difficult to ascertain a consistent pattern of lifestyle changes among children and adolescents. Generally, the trends of lifestyle changes reported in this study during the period of schools’ closure were found to be going in a health-compromising direction. Although we noticed an increase in fruit intake during closure compared to before, this increase was not statistically significant. On the other hand, the other adverse dietary changes including the reduction in vegetable intake, and the increase in unhealthy diets such as soft drinks, fried food, junk food, and sweets were all significant. Schools’ closure was among many other restrictive measures implemented by the country to contain the spread of COVID-19 including the closure of malls, gyms, and play areas, and limiting social gatherings. Such restrictive measures may have affected normal food-related practices. Many people tended to limit their going out of their homes even to the grocery shops out of fear of catching the infection and tended to buy more processed or canned food which tend to be high in fats, sugars, and salt because it was easier to store [1]. This might have affected the dietary patterns and behaviors of children since parents are responsible for buying and preparing food for their children whose intake of particular foods is influenced by the types of foods present at home [24].

We found a statistically significant reduction in the intake of vegetables during school closures contrary to other studies that reported an increase in vegetable intake [11, 25]. The nonsignificant increase in fruit intake matches the results of a study in Italy [25]. On the other hand, another study in Italy with a longitudinal design and another one in Jordan managed to detect a significant increase in fruit intake [6, 26]. We found significant increases in the intake of soft drinks/sweetened beverages, fried food, junk food, and sugar-based sweets like candies, Jam …. consistent with the results of other studies conducted in the region and worldwide [6, 12, 26]. Saudi Arabia reported that 39.4% of children and adolescents had difficulty maintaining a healthy, balanced diet during the pandemic, including an increase in the consumption of simple carbohydrates, fried foods, and soft drinks [12]. Similarly, 45.6% of children and adolescents in a study in Egypt reported increased sweets and unhealthy food consumption after COVID-19-related closure [4]. In the same context, studies conducted in several European countries reported a significant increase in the consumption of fried food and an increase in the proportion of adolescents consuming sweet food every day from 14% to over 20% during lockdown measures [27]. Staying at home for prolonged periods increase the feeling of boredom which has been associated with a greater energy intake, as well as the consumption of higher quantities of fats, carbohydrates, and proteins [28]. The stress brought about by the pandemic and its related containment measures has pushed people toward overeating, mostly for sugary “comfort foods” [29, 30]. Emotional eaters tend to deal with their feelings by turning to high energy and low nutrient density foods [31]. Although the emotional eating behaviors among children have been rarely investigated, some studies highlighted the interrelationship between stressful events, negative emotions, and emotional eating among children, with positive associations observed between problems and the consumption of sweets and fat-rich food [32]. In a study conducted in France to assess children and parenting behaviors during lockdown measures, emotional eating was increased in both [11]. Emotion regulation which is defined as a set of conscious or unconscious behaviors, skills, and strategies that can modulate one’s emotional expression has been extensively investigated in obesity research [31]. Evidence has shown strong associations between poor emotional regulation and the risk of obesity among children which is mediated by emotional eating [33]. Given these associations, further research is needed to better understand the predictors and determinants of emotional eating among children and adolescents. Unlike other studies, we found a significant increase in junk/fast food intake among children and adolescents during closure. Moreover, the average frequency of fast food intake in this study (1–2 times/ week) was higher than was reported in other countries in Europe and Latin America (0.2–0.8 times/week) [34]. However, our result does not match a previous study conducted in Qatar that assessed food behavior and consumption patterns early in the pandemic (May- June 2020) and reported that about one-third of participants reduced their ordering of fast food during the pandemic [35]. This might be explained by the time of conducting that study earlier in the pandemic contrary to ours. It is expected that early in the pandemic when many uncertainties were surrounding the virus and its modes of transmission, people were more cautious, afraid of catching the infection and hence more willing to comply with physical distancing measures and minimized contact with others, and consequently, they tended to cook at home more, so the ordering from fast food restaurants was expected to be less [1]. With time, when such uncertainties were revealed, and with the availability of COVID-19 vaccines, people began to reverse back to a normal state, but the stress associated with the prolonged restrictive measures pushed people more toward comfort foods as mentioned earlier leading to more junk food intake. This study showed that lower parental educational levels were somehow protective against adverse dietary changes compared to higher levels. Children of parents with university degrees or higher were more likely to increase their intake of unhealthy food such as sugar, added drinks, fried food, fast food, and sweets compared to children of parents with lower educational levels. This might look like contradicting existing evidence from other studies conducted before the pandemic where lower parental educational levels were associated with a higher intake of unhealthy food [36, 37]. But in fact, it is not. Here, the association was with the change in the intake (the difference between the two time periods). To support this, we compared the intake of such unhealthy food groups between different parental educational levels before and during closure and found that it was significantly higher with lower educational levels during both periods compared to higher levels. Having a better baseline intake profile among students whose parents had higher educational levels might explain why the change (increase in intake) was more obvious among them during closure. We found that employed mothers were more likely to report adverse dietary changes among their children with increased consumption of unhealthy foods. During schools’ closure, mothers had to deal with homeschooling their children which is stressful and time-consuming mainly for working mothers [38]. Overloading employed mothers with homeschooling tasks might have further limited their time and abilities to prepare healthy meals for their children resulting in the observed change in this study. The literature showed that maternal employment was associated with spending less time in meal preparation at home and less time eating with their children [39]. Being away from home, the supervisory role of the employed mothers on their children’s diet becomes limited leading them to adopt unhealthy dietary habits and rely more on processed food, ready-made meals, and meals prepared at schools if any [40]. This highlights the importance of establishing healthy canteens in schools that serve balanced healthy meals for students and staff. In Qatar, children at all of Qatar Foundation’s schools (which are private schools) are being served the healthiest meals possible during their lunch breaks as part of a healthy school canteen program [41]. However, this program is not yet implemented in governmental schools in Qatar where many students go. Considering this, more emphasis should be given to providing the necessary awareness to parents of students at governmental schools on how to prepare healthy meals for their children. The reduction in physical activity including active play during schools’ closure shown in this study is supporting the results of other studies conducted regionally [6, 8, 14, 15], and globally [5, 9, 42–47]. Younger children were found to be more physically active before and during closure than older ones. Moreover, parents of younger children were less likely to report a reduction in their children’s physical activities. One explanation might be that we included the active play in the assessment of physical activity, and it is expected that active play is more common in younger children than teens and teenagers which might have contributed to this result. In addition, active play is less likely to be affected by the COVID-19 restrictive measures than other types of physical activity like walking, cycling, and swimming that require going out of the home. One study showed that the most common types of children’s physical activity reported by parents during COVID-19 were free play/unstructured activities like running around [48]. Policymakers might need to focus their efforts on teens and teenagers when implementing physical activity-related lifestyle interventions. Students with chronic diseases like asthma were more likely to report a reduction in physical activity. This is expected as having a history of chronic diseases, particularly asthma is associated with a greater risk of catching COVID-19 and developing severe disease. Parents of asthmatic children most probably were more cautious and more reluctant to allow their children to go out for physical activity where they might catch the infection. Exercise can also exacerbate asthma and lead to exercise-induced bronchoconstriction, particularly among children and adolescents, an undesirable consequence, especially in light of the COVID-19 pandemic [49]. We also noticed that the reduction in physical activity was more probable among non-Qatari children compared to Qataris. One explanation might be that many Qatari children live in big houses with private swimming pools, large backyards, and sometimes, a personal gym. These factors can facilitate maintaining the physical activity of Qatari students compared to non-Qatari. The unfavorable changes in the lifestyle of children and adolescents might have put them at risk of gaining weight which might further increase their risk of non-communicable diseases.

### Strengths and Limitations

This study provided some insights into the impact of COVID-19-related school closures on two important aspects of the lifestyle of children and adolescents in Qatar which are diet and physical activity and highlighted the sociodemographic predictors of the adverse changes. Conducting this study at a national level by taking a sample from all students registered at the governmental schools in Qatar helped us achieve an acceptable sample size (1546) with a good response rate of about 46%. We validated the data collection tool (face, content, and translational) as described above. This study is one of the few conducted in the Middle East to address lifestyle changes among children and adolescents during COVID-19-related home confinement measures. We used a stratified random sampling technique using nationally trusted sampling frames and did not rely on convenient sampling like other studies. Despite these strengths, some limitations need to be acknowledged. The self-reporting of the data from the parents’ perspectives might have introduced some recall and social desirability bias. Moreover, we assessed the changes in lifestyle aspects by basing the questions on two time periods (before and during) retrospectively and this increases the possibility of recall bias.

## Conclusion

The trends of lifestyle changes reported in this study during the periods of COVID-19-related school closures were found to be going in a health compromising direction. Important predictors for different adverse changes included sex, the child’s developmental stage, parental educational levels, and maternal employment. The School Health Services and Programs in Qatar which provides clinical and preventive health services to students at governmental schools under the umbrella of the Primary Health Care Corporation needs to collaborate with policymakers and other stakeholders including students, parents, and teachers to implement effective lifestyle related interventions. Further research is needed to explore the persistence of such adverse lifestyle changes in the post-pandemic era when restrictive measures were eased.

## Supplementary Information

Below is the link to the electronic supplementary material.

Supplementary file1 (DOCX 37 KB)

## Data Availability

The data that support the findings of this study are available from the corresponding author upon reasonable request.

## Abbreviations

HMC: Hamad Medical Corporation
PHCC: Primary Health Care Corporation
