# Supplementary material for: A Sociodemographic Analysis of the Impact of COVID-19-Related Schools’ Closure on the Diet and Physical Activity of Children and Adolescents in Qatar
Source: J Epidemiol Glob Health. 2023 May 4;13(2):248–65. doi: 10.1007/s44197-023-00101-8 (PMC10157550; doi:10.1007/s44197-023-00101-8)
Supplement: Supplementary file 1 — Supplementary file1 (DOCX 37 KB) [file 44197_2023_101_MOESM1_ESM.docx]

**The English version of the questionnaire**

| **Section A**: **Socio-demographic characteristics and background information:** | | | | | | | |
| --- | --- | --- | --- | --- | --- | --- | --- |
| **Question** | **Choices:** | | | | | | |
| 1. Age |  | | | | | | |
| 1. Age of the mother (in years) |  | | | | | | |
| 1. Age of the father (in years) |  | | | | | | |
| 1. Nationality of the child |  | | | | | | |
| 1. School grade of the child |  | | | | | | |
| 1. Highest degree of education for mother? | No formal education | primary school level | | preparatory school level | | Secondary/ high school level | College or higher |
| 1. Highest degree of education for Father? | No formal education | primary school level | | preparatory school level | | Secondary/ high school level | College or higher |
| 1. Number of child siblings |  | | | | | | |
| 1. Family history of obesity or overweight? | Yes, specify who?? | | No | | | | |
| 1. Mother employment status | Employed | | Not employed | | | | |
| 1. Does the child suffer from any chronic diseases (like DM, Asthma…) | Yes (specify) | | | | No | | |

| **Section B: Diet** | | | | | | | | | | | | | | |
| --- | --- | --- | --- | --- | --- | --- | --- | --- | --- | --- | --- | --- | --- | --- |
| 1. How many servings of fruit did your child usually eat in a day? (**A one servings of fruits equals to 1 medium fruit (e.g., banana, apple, orange), or 2 small (Kiwi, apricot) 1 cup sliced fruit** **(e.g., watermelon) or canned fruit, or ½ cup 100% fresh juice, or ¼ cup dried fruit)** *Before school closure* | Less than 1 serving/day | | | 1-2 servings per day | | | more than 2 servings /day | | | | | | | |
| 1. How many servings of fruit did your child usually eat in a day? *During school closure* | Less than 1 serving/day | | | 1-2 servings per day | | | more than 2 servings /day | | | | | | | |
| 1. How many servings of vegetables did your child usually eat in a day? **A one servings of vegetables equals to ½ cup of cooked vegetables or 1 cup of salad vegetables (raw green leafy vegetables) or ½ medium of starchy vegetables (potato) or 1 medium vegetables (cucumber, carrot).** *Before school closure* | Less than 1 serving/day. | | | 1-3 servings/day | | | 4-6 servings/day | | | More than 6 servings /day). | | | | |
| 1. How many servings of vegetables did your child usually eat in a day? *During school closure* | Less than 1 serving/day. | | | 1-3 servings/day | | | 4-6 servings/day | | | More than 6 servings /day). | | | | |
| 1. How much soft drinks, sweetened beverages, energy drinks or sports drinks did your child usually drink? (**One can of soft drink = 1 ½ cups)** *Before school closure* | My child does not drink these drinks | | < 1 cup/week | | | 1-3 cups/week | | 4-6 cups/week | | | 1-2 cups/day | | 3 or more cups/day | |
| 1. How much soft drinks, sweetened beverages, energy drinks or sports drinks did your child usually drink? (*During school closure* | My child does not drink these drinks | | < 1 cup/week | | | 1-3 cups/week | | 4-6 cups/week | | | 1-2 cups/day | | 3 or more cups/day | |
| 1. How often did your child eat fried food (French-fried potatoes, fried chicken….) prepared at home? *Before school closure* | never or rarely | less than once a week | | | About 1 to 2 times a week | | About 3 to 4 times a week | | About 5 to 6 times a week | | | about once a day | | 2 or more times a day |
| 1. How often did your child eat fried food (French-fried potatoes, fried chicken….) prepared at home? *During school closure* | never or rarely | less than once a week | | | About 1 to 2 times a week | | About 3 to 4 times a week | | About 5 to 6 times a week | | | about once a day | | 2 or more times a day |
| 1. How often did your child eat junk food (burger, pizza…) from fast food restaurants? *Before school closure* | never or rarely | less than once a week | | | About 1 to 2 times a week | | About 3 to 4 times a week | | About 5 to 6 times a week | | | about once a day | | 2 or more times a day |
| 1. How often did your child eat junk food (burger, pizza…) from fast food restaurants? *During school closure* | never or rarely | less than once a week | | | About 1 to 2 times a week | | About 3 to 4 times a week | | About 5 to 6 times a week | | | about once a day | | 2 or more times a day |
| 1. How often did your child eat sugar-based sweets/ sweets (candies, chocolate, jam, Nutella...)? *Before school closure* | never or rarely | less than once a week | | | About 1 to 2 times a week | | About 3 to 4 times a week | | About 5 to 6 times a week | | | about once a day | | 2 or more times a day |
| 1. How often did your child eat sugar-based sweets/ sweets (candies, chocolate, jam, Nutella...)? *During school closure* | never or rarely | less than once a week | | | About 1 to 2 times a week | | About 3 to 4 times a week | | About 5 to 6 times a week | | | about once a day | | 2 or more times a day |

| **Section C: Physical Activity** | | | | | | | | |
| --- | --- | --- | --- | --- | --- | --- | --- | --- |
| 1. In a typical week, on how many days have your child done a total of 60 minutes or more of physical activity, which was enough to raise his/her breathing rate? This may include sport, exercise and brisk walking, cycling for recreation or to get to and from places or active playing’ (*Before school closure.)* | 0 | 1 | 2 | 3 | 4 | 5 | 6 | 7 |
| 1. In a typical week, on how many days have your child done a total of 60 minutes or more of physical activity, which was enough to raise his/her breathing rate? This may include sport, exercise and brisk walking, cycling for recreation or to get to and from places or active playing’ (*During school closure.)* | 0 | 1 | 2 | 3 | 4 | 5 | 6 | 7 |
| 1. Did your child use to practice any type of sports on regular bases outside the school? *Before school closure.* | Yes | | | | No | | | |
| 1. Did your child use to practice any type of sports on regular bases outside the school? *During school closure.* | Yes | | | | No | | | |
